# Impact of rescanning and normalization on convolutional neural network performance in multi-center, whole-slide classification of prostate cancer

**DOI:** 10.1038/s41598-020-71420-0

**Published:** 2020-09-01

**Authors:** Zaneta Swiderska-Chadaj, Thomas de Bel, Lionel Blanchet, Alexi Baidoshvili, Dirk Vossen, Jeroen van der Laak, Geert Litjens

**Affiliations:** 1 Department of Pathology, Radboud University Medical Center, Nijmegen, The Netherlands; 2 Digital and Computational Pathology, Philips, Best, The Netherlands; 3 Laboratorium Pathologie Oost-Nederland, LabPON, Hengelo, The Netherlands; 4 Center for Medical Image Science and Visualization, Linköping University, Linköping, Sweden

**Keywords:** Cancer, Computational biology and bioinformatics, Medical research

## Abstract

Algorithms can improve the objectivity and efficiency of histopathologic slide analysis. In this paper, we investigated the impact of scanning systems (scanners) and cycle-GAN-based normalization on algorithm performance, by comparing different deep learning models to automatically detect prostate cancer in whole-slide images. Specifically, we compare U-Net, DenseNet and EfficientNet. Models were developed on a multi-center cohort with 582 WSIs and subsequently evaluated on two independent test sets including 85 and 50 WSIs, respectively, to show the robustness of the proposed method to differing staining protocols and scanner types. We also investigated the application of normalization as a pre-processing step by two techniques, the whole-slide image color standardizer (WSICS) algorithm, and a cycle-GAN based method. For the two independent datasets we obtained an AUC of 0.92 and 0.83 respectively. After rescanning the AUC improves to 0.91/0.88 and after style normalization to 0.98/0.97. In the future our algorithm could be used to automatically pre-screen prostate biopsies to alleviate the workload of pathologists.

## Introduction

Prostate cancer is the most common cancer in men and the third most common tumor type worldwide^1,2^. In 2018, 1.3 million new cases have been diagnosed (7.1% of all diagnosed cancers), and 28% of these patients died as a result of the disease^1^. Prostate cancer is typically diagnosed through ultrasound-guided biopsy after initial suspicion has arisen through, for example, a prostate specific antigen (PSA) blood test. During the prostate biopsy procedure, 6–12 core samples are taken from a patient^3^ resulting worldwide in more than 15 million specimens annually, which is expected to increase further with the aging of the population. All these specimens have to be evaluated by pathologists. However, in many countries there is a lack of pathologists which is only expected to increase in the years to come. Automating (part of) the evaluation of prostate biopsies might help mitigate the lack of clinical pathology.

The histopathological analysis could be streamlined significantly if these negative slides (i.e. slides without pathology) could automatically be excluded without expelling any slides containing cancer. Significant progress has been made in this respect, revealing the huge potential of deep learning (DL) methods^4–6^. In histopathology, deep learning based algorithms have been used to solve a variety of tasks, such as mitotic Figure detection^7^, lung adenocarcinoma segmentation^4^, glomeruli detection^8^ or tissue analysis in colorectal cancer^9^.

**Figure 1:**
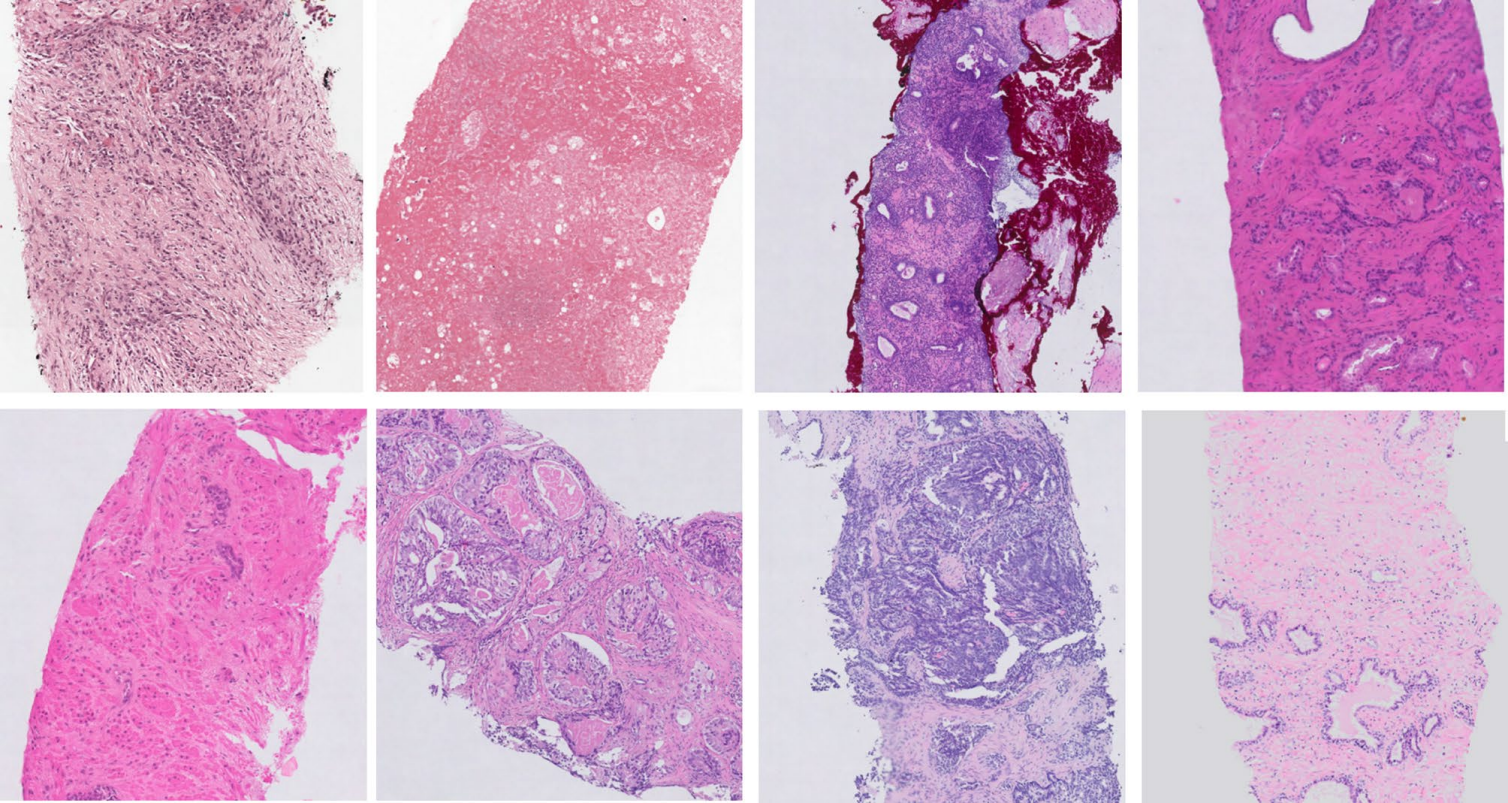
Example of the heterogeneity in appearance in prostate biopsy specimens for slides from the development set. The figure was created using ASAP^34^ software, ver. 1.9.0 https://github.com/computationalpathologygroup/ASAP.

However, histological slides from different institutions show heterogeneous appearance as a result of the different preparation and staining procedures (different colors, intensity, saturation) (Fig. 1). As a result, there is a high probability that a model trained on data from one medical center may not be applicable to slides from another center. The key challenge is to develop a system robust to a variety of biological, staining or scanning settings.

In this study, we present work on automatic prostate cancer detection through a method developed on a multi-center dataset including 582 manually annotated slides. We investigated the impact of scanning systems on deep learning algorithms performance. To this end, we re-scanned two independent sets on the same scanner that was used to digitize the development set. Additionally, we proposed a cycle-GAN style normalization as a way to improve method robustness. We compare two different normalization approache (color and style normalization) and investigated their impact.

### Related work

In last years, we observe a growing interest in the application of DL systems to support prostate cancer evaluation. In literature several related studies on prostate cancer were published^10–14^. Two main tasks can be distinguished: (a) cancer detection and segmentation, and (b) Gleason grading.

In 2014, Cruz-Roa et al.^15^ were among the first to apply deep learning to whole-slide images in the context of breast cancer. In 2015, Litjens et al.^16^ proposed the application of DL to detect prostate cancer on whole-slide images. The limitation of that work was the use of a training data set from a single medical institution. This is a key issue due to the large stain (color, intensity) variability between centers. Due to this, a neural network trained on data from a single center can potentially poorly generalize to data from other centers. This can partially be alleviated with data augmentation, but most likely not fully. A tool to support pathologists’ work should be robust on this type of variances. Arvidsson et al.^12^ proposed an auto-encoder application to prostate cancer detection. They achieved good results, with accuracy  88% on an independent data set of 39 whole slide images (WSIs). However, this method was evaluated only at the patch level. The transition from patch level classification to a whole-slide level classification is challenging. Burlutskiy et al.^13^ present an innovative approach to detect healthy glands in a WSI image, which allows detecting potentially cancerous areas. However, the basic assumption that glands not detected as healthy are cancerous does not hold, especially given the wide range of gland in clinical practice. Khan et al.^14^ showed that transfer learning based on the same domain can improve final segmentation results. They present decent results with an area under the curve (AUC) of 0.924 at the patch level. However, their test set is small and includes only 6 slides, originating from the same distribution as slides used for network fine-tuning. The most recent work on automatic prostate cancer detection is work presented by Campanella et al.^17^, which is characterized by using a large dataset includes several thousands of slides collected in one medical center. The applied dataset allowed authors to use the scale effect and develop a using a multiple-instance-learning (MIL) approach. In the paper, the authors present very high performance (AUC = 0.99 for the test set and AUC = 0.93 for external test set), where the small gap between the internal and external test set AUC shows a good generalization performance of the method. However, it should be noted that collecting several thousands of slides is not trivial for many tasks and in some cases impossible. In the current study we investigate whether we achieve similar results with a much smaller supervised dataset, where both our training and testing data are multi-center or digitized on scanners from multiple vendors. Specifically, we will assess whether multi-center training data results in improved generalization performance. We cannot exactly compare the performance of the two methods due to the different datasets. However, given the fact that the data from Campanella et al. is not publicly available, performing a similar analysis is the best we can do. Automatic Gleason grading, which is also a popular area of research^10,11^, was not studied in this paper.

### Our contribution

The main goal of this study is to investigate the robustness of convolutional neural networks to stain and scanning variability for automatic detection of prostate cancer in WSIs and the effect of rescanning and normalization. This paper has four main contributions: (I) we developed and compared different deep learning approaches that address prostate cancer detection at whole-slide image-level based on a multi-center dataset, (II) the proposed method was evaluated based on two independent datasets of 85 and 50 whole-slide images digitized on scanners from two vendors and from a medical center not included in the development set, (III) we took into account the influence of scanner variability on a deep learning classification results, and (IV) we investigated the influence of color and style normalization on classification results.

## Results

### Experimental setup

In this work, four experiments were conducted to evaluate method performance. In the first experiment, U-Net, DenseNetFCN and EfficientNet performance were compared on development set in cross-validation. For all networks, the same training and validation data were used. The total development set consisted of 582 WSIs. 486 WSIs were used in a three-fold cross-validation procedure for network training, and 96 WSIs were kept separate to optimize post-processing hyperparameters. The 486 whole-slide images in the development set were divided into: training (n = 264), validation (n = 60) and test set (n = 162), were in each of group ~ 25% cases contained cancer. During training, the validation loss was monitored to determine when to stop training. The Dice coefficient metric at the patch-level was monitored for DenseNetFCN and U-Net, and the accuracy for EfficientNet. Training was repeated for each fold with test set results merged after all folds were completed. This results in a tumor likelihood map for every case in the entire development set.

In the second experiment we retrained the best model on the whole development set (training WSIs: 402, validation WSIs: 84) and evaluated it on the two independent test sets, using the hyperparameters for both network training and post-processing as obtained in experiment 1.

In the third experiment, the effect of scanning variability was investigated. Slides from both independent test sets were re-scanned on the scanner used to digitize the development set (Table 3). The developed algorithm was applied to the re-scanned slides to analyze the effect of scanning variation.

The fourth experiment investigates the influence of a normalization procedure on the method performance. Slides from the independent test sets were normalized using two different approaches: (a) color normalization to the color domain of the development set and (b) color and style normalization using a cycle-GAN network. The developed algorithm was applied on normalized slides to investigate the effect of normalization.

For all experiments a slide-level likelihood was obtained for each case which was used to construct a receiver-operating characteristic (ROC) curve and calculate the area under the curve (AUC) in addition to several sensitivity/specificity pairs and overall accuracy. Bootstrapping was used to obtain ROC confidence intervals^18^. The bootstrap was performed by sampling with a replacement on the prediction indices, and a number of bootstraps was 2000.

### Experimental results

In this section, we report the quantitative results of four performed experiments. Results for each experiment are presented in independent subsections.

### Experiment I: three-fold cross-validation

**Figure 2:**
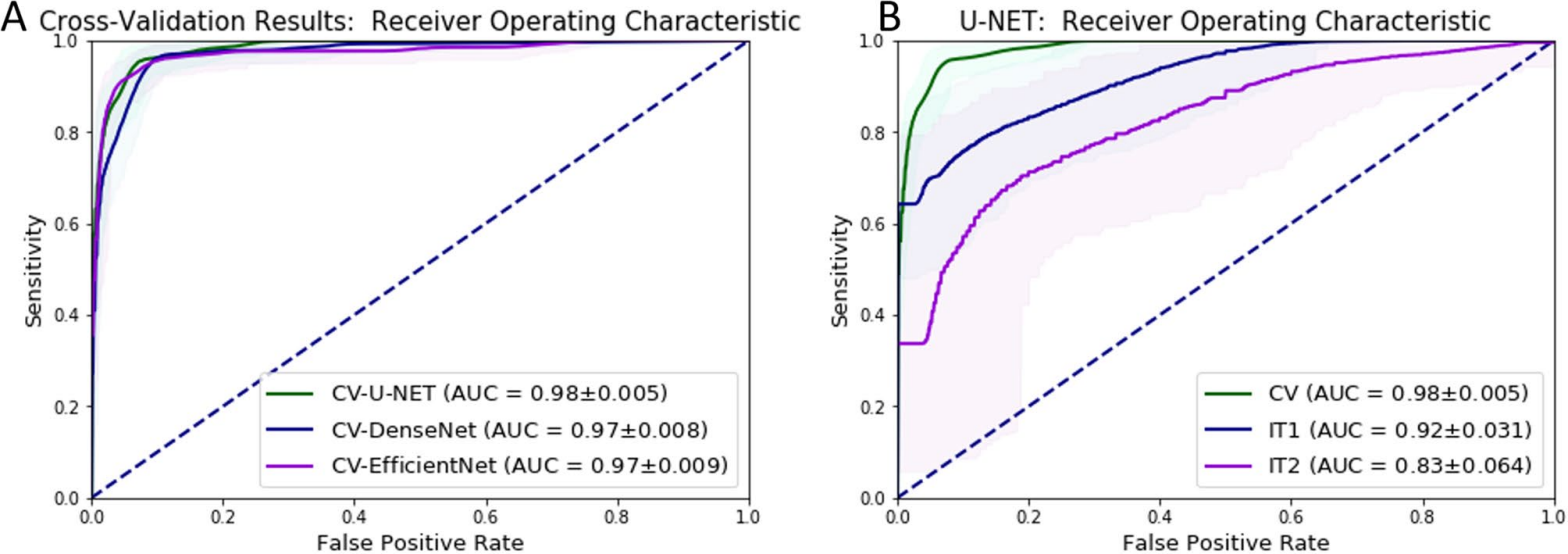
ROC curves for binary whole-slide classification, where: (**A**) the results for the three-fold cross validation for each network, (**B**) the results for the best model (U-Net), CV—results for the three-fold cross validation (green), IT1—results for the independent test set I, IT2—results for the independent test set II, ±—confidence interval obtained through bootstrapping.

The average patch level classification results in terms of Dice coefficients after training was 0.80 (the Jaccard index: 0.67) and 0.74 (the Jaccard index: 0.59) for U-Net and DenseNetFCN respectively. Next, we analyzed the classification results at the whole-slide level, where each slide is labeled as either cancer or normal. In Table 1 and in Fig. 2 we present the detailed results of the ROC analysis on the full development set, showing that the AUC for U-Net is higher than for DenseNetFCN and EfficientNet.

### Experiment II: independent test sets

**Figure 3:**
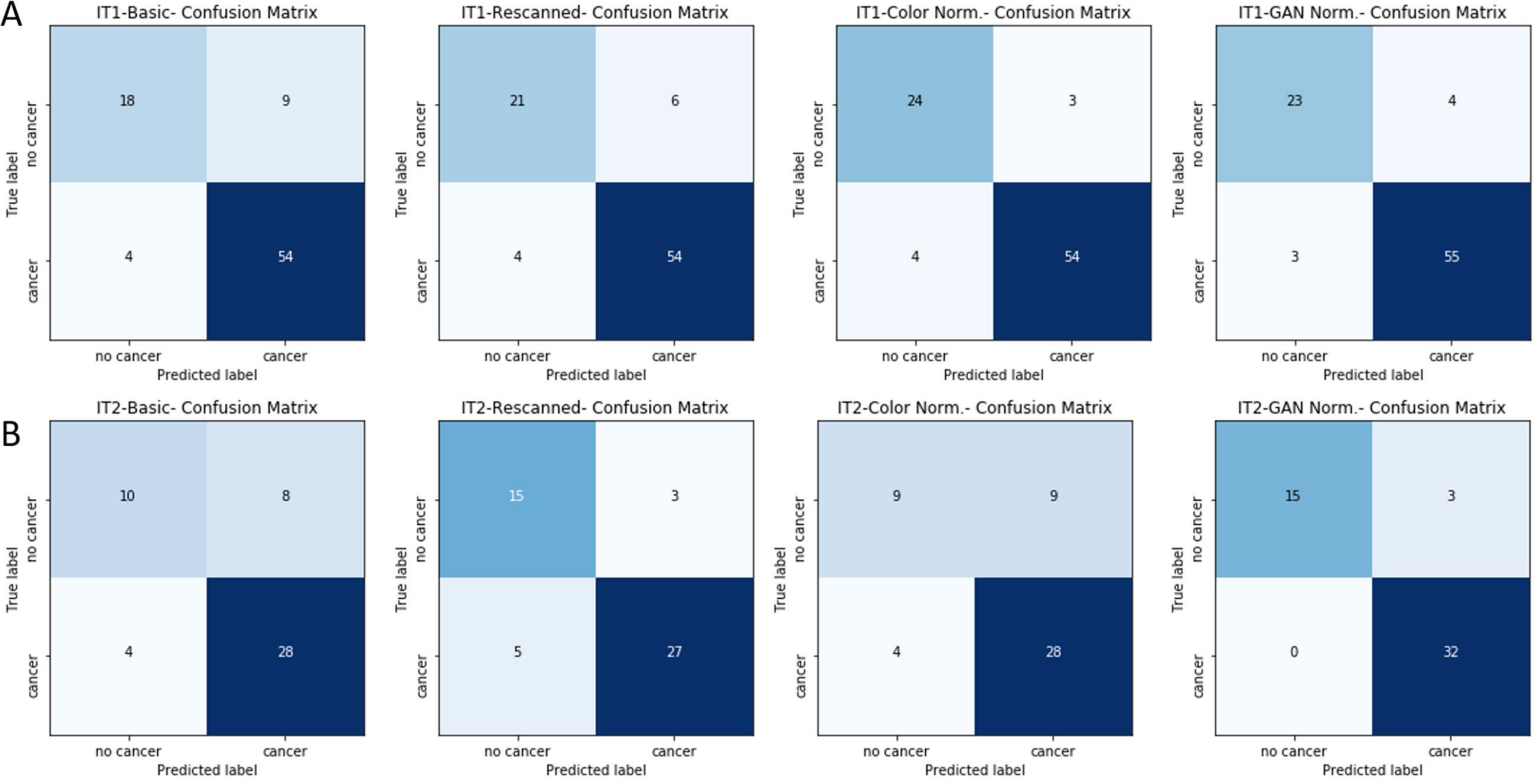
The Confusion Matrixs on the whole-slide level for the independent test sets.

Given the better performance, further experiments where conducted with the U-Net architecture. The U-Net architecture was trained using the full development set (1,246,629 training patches, and 221,576 validation patches). Next, the method was evaluated using two independent test sets. The results of the ROC analysis of this retrained architecture are shown in Table 2 and in Figs. 3, 5 and 6.

**Table 1:** Three-fold cross-validation results on a slide level.

Model	Point	Average: cross validation (CV)				IT1				IT2			
		SE	SP	ACC	AUC	SE	SP	ACC	AUC	SE	SP	ACC	AUC
U-Net	P1	1	0.75	0.82	0.98 ± 0.05	1	0.41	0.81	0.92 ± 0.03	1	0.05	0.66	0.83 ± 0.06
	P2	0.5	1	0.87		0.64	1	0.75		0.33	1	0.57	
	P3	0.85	0.97	0.94		0.92	0.65	0.83		0.88	0.53	0.75	
DenseNetFCN	P1	1	0.41	0.56	0.97 ± 0.08	–	–	–	–	–	–	–	–
	P2	0.31	1	0.82		–	–	–	–	–	–	–	–
	P3	0.91	0.93	0.92		–	–	–	–	–	–	–	–
EfficientNet	P1	1	0.28	0.47	0.97 ± 0.09	–	–	–	–	–	–	–	–
	P2	0.36	1	0.84		–	–	–	–	–	–	–	–
	P3	0.89	0.97	0.95		–	–	–	–	–	–	–	–

Results are presented for three points of the ROC curve: P1—specificity equal 1, P2—sensitivity equal 1, P3—the best accuracy, where: SE—sensitivity, SP—specificity, ACC—accuracy.

**Table 2:** Performance on the whole-slide level on the independent test sets.

Test set	Point	A-Basic				B-Rescanned				C-Color norm.				D-GAN style norm.			
		SE	SP	ACC	AUC	SE	SP	ACC	AUC	SE	SP	ACC	AUC	SE	SP	ACC	AUC
IT1	P1	1	0.41	0.81	0.92 ± 0.03	1	0.3	0.78	0.91 ± 0.04	1	0.72	0.9	0.96 ± 0.03	1	0.5	0.84	0.98 ± 0.01
	P2	0.64	1	0.75		0.42	1	0.60		0.74	1	0.82		0.74	1	0.86	
	P3	0.92	0.65	0.83		0.93	0.73	0.87		0.93	0.87	0.91		0.96	0.87	0.93	
IT2	P1	1	0.05	0.66	0.83 ± 0.06	1	0.11	0.68	0.88 ± 0.05	1	0.05	0.65	0.81 ± 0.07	1	0.75	0.91	0.97 ± 0.03
	P2	0.33	1	0.57		0.53	1	0.7		0.21	1	0.49		0.69	1	0.80	
	P3	0.88	0.53	0.75		0.85	0.75	0.81		0.91	0.50	0.76		0.99	0.80	0.92	

Results are presented for three points (thresholds) of the ROC curve: P1—specificity equal 1, P2—sensitivity equal 1, P3—the best accuracy, where: A—original slides, B—rescanned slides, C—color normalized slides, D—style normalized slides by cycle-GAN method, SE—sensitivity, SP—specificity, ACC—accuracy

### Experiment III: effect of scanner variability

**Figure 4:**
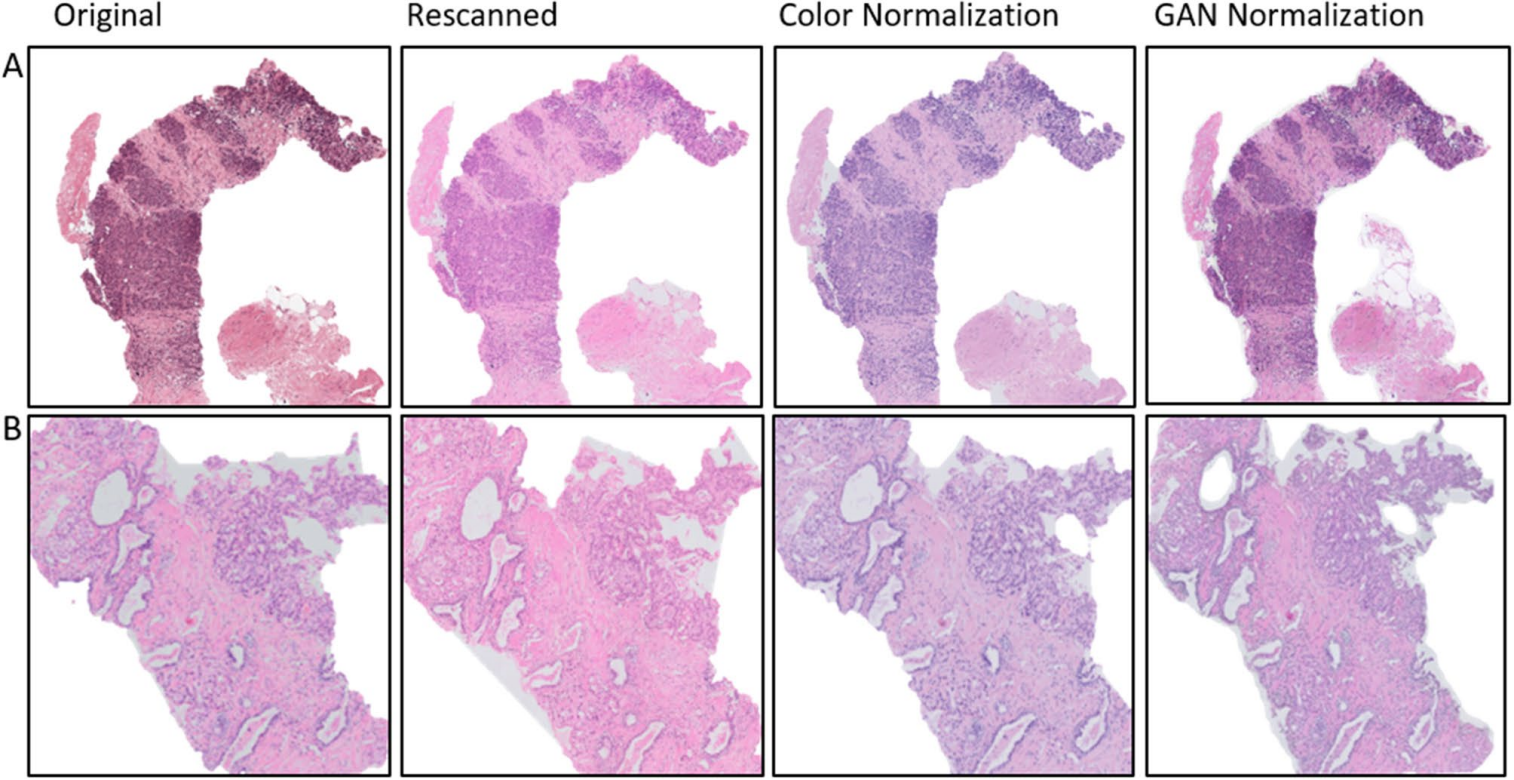
Example of re-scanned specimens and color normalized slides for (**A**) IT1 and (**B**) IT2.

**Figure 5:**
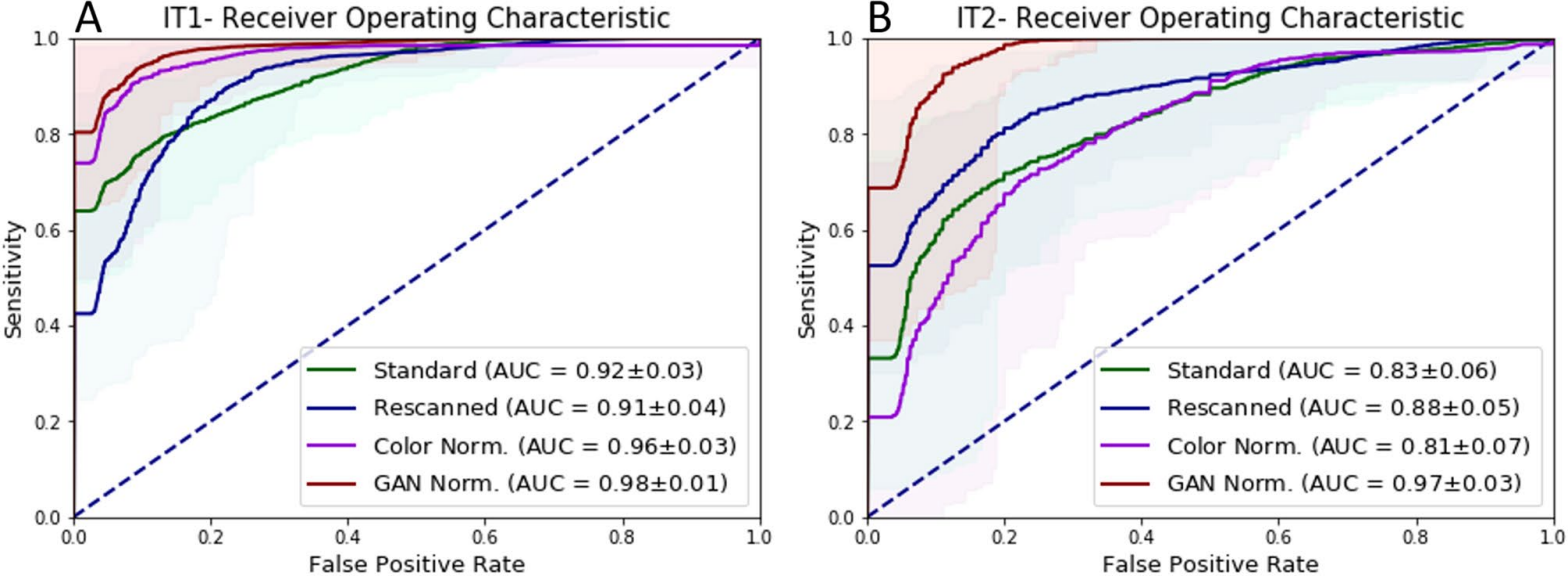
ROC curve- Re-scanning and color normalization performance. The comparison of ROC curve results for original, re-scanned, color normalized specimens and GAN normalized specimens for two independent test sets, where: (**A**) results for the independent test set I, (**B**) results for the independent test set II, ±—confidence interval obtained through bootstrapping.

**Figure 6:**
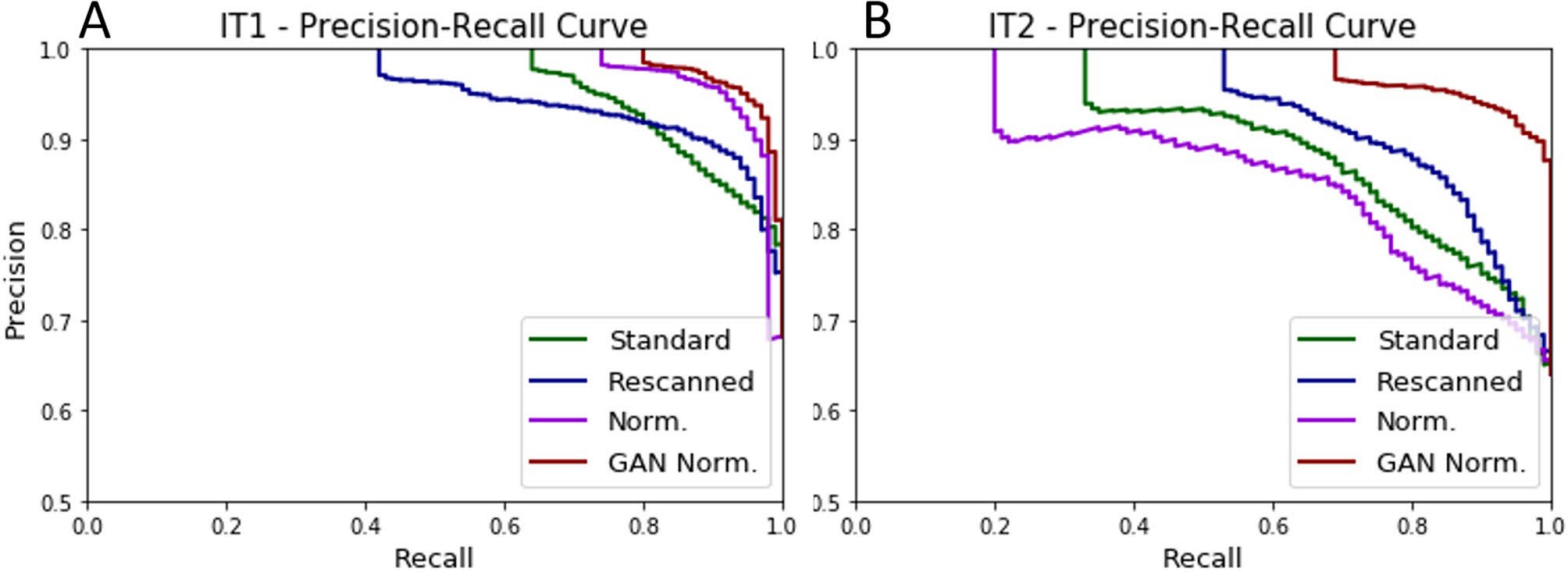
Precision-Recall curve: Re-scanning and color normalization performance. The comparison of Precision-Recall curve results for original, re-scanned, color normalized specimens and cycle-GAN normalized specimens for two independent test sets, where: (**A**) results for the independent test set I and (**B**) results for the independent test set II.

In the following experiment, the independent test sets were re-scanned on the Philips Ultrafast scanner. The re-scanning procedure allows to remove one source of variability, allowing us to assess the performance differences caused by scanner differences. In Fig. 4 we show the same slides before and after re-scanning, where we can easily observe significant differences in color representation, that are a result of the scanning system. In Table 2B and in Figs. 3, 5 and 6 we presented detailed results before and after re-scanning.

### Experiment IV: color and style normalization

**Figure 7:**
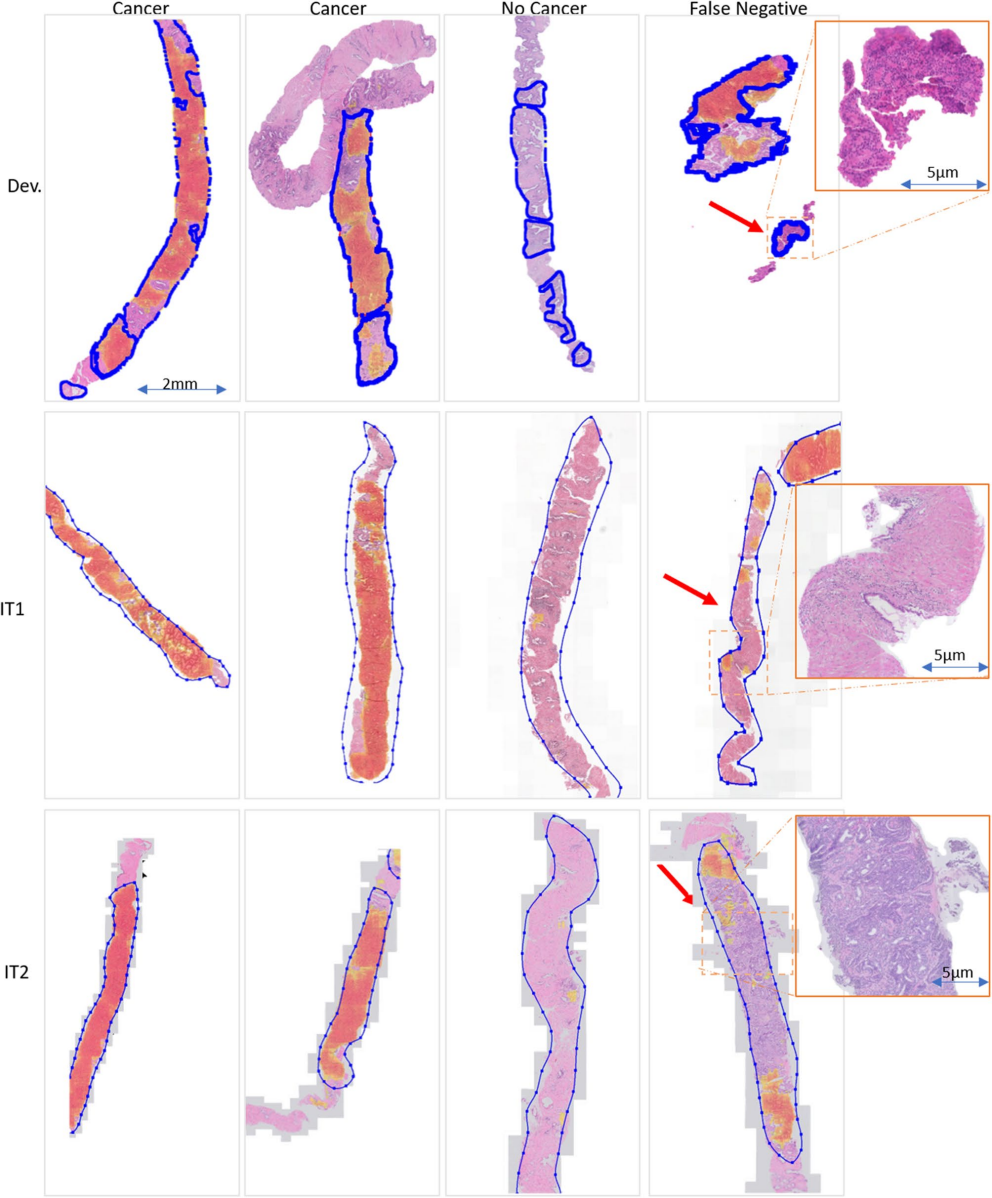
Example of graphical results for GAN-normalized specimens with cancer probability map for positive cases (cancer), negative cases (no cancer) and areas with false negative detection, where: Dev.—results for the development data set, IT1—independent test set 1, IT2—independent test set 2, blue line—annotations, red arrow—not detected areas (false negative areas). The figure was created using ASAP^34^ software, ver. 1.9.0 https://github.com/computationalpathologygroup/ASAP.

In the last experiment, the influence of color normalization (stain normalization) on the final classification result was investigated. To do this, the color normalization procedure developed by Ehtseshami Bejnordi et al.^19^ was applied on both independent test sets. An example of color normalization is presented in Fig. 4. Detailed results are included in the Table 2C and Figs. 5 and 6 whereas graphical results are presented in Figs. 3 and 7.

## Discussion

The results in this work highlight the potential for deep learning systems to be used as a triage tool, where at very high sensitivity ($>\,0.99$), a large number of normal slides would not have to be checked by an expert. This even holds when looking at our results on the independent test sets, albeit with slightly lower specificity than on the development set. This highlights there is still room for future improvements, especially in the case of robustness to center variability.

Our results, analog to those presented by Campanella et al.^17^, show that even with extensive data-augmentation performance of deep learning algorithms deteriorate on data from different institutions and scanning systems, even as high as 15%. Thus, the strategy used in many papers where data from a single institution is used will result in positively biased performance metrics, even in the case of correctly splitting the data in training, validation and test sets. Ideally, every paper should include results on independent test sets coming from a different institution.

A key strength of the presented study is the use of a multi-center cohort, scanned with different scanners. This allowed us to increase method robustness. Annotations used for the training were prepared by multiple experts, which includes inter-observer variability in the training set, allowing the method to adapt to different styles of annotating. In a case of used annotations prepared by a single expert, the network could overfit to that one expert.

Analysis of the patch level validation performance shows that semantic segmentation methods (U-Net and DenseNetFCN) achieved Dice coefficient metrics in a range of 0.74–0.80 (the Jaccard index in a range of 0.59–0.67), whereas EfficientNet achieved an accuracy of 0.70. This shows that both approaches, semantic segmentation and patch classification, are able to detect tumor areas. Analysis of the three-fold cross-validation results for all networks shows good results with AUC in range 0.97–0.98, where slightly higher results were achieved by the U-Net. One advantage of the EfficientNet architecture is that it is lightweight: the size of a trained model is 48 MB, whereas the size of the trained U-Net model is 237 MB.

Analysis of results of Experiment 1 (Fig. 2 and Table 1) present an excellent result for the test set from the same institution (AUC in a range 0.97–0.98) and lower results for both independent test sets. As such, extensive data augmentation alone is not enough to ensure that algorithm performance generalizes. However, the overall performance drop on IT1 is reasonable, with a loss of $\sim$ 6%, slightly less than reported in^17^. For IT2 the drop is higher with $\sim$ 15%, which would be unacceptable for adoption in clinical practice. These drops can be caused due to a variety of reasons, such as different scanners, tissue preparation and staining procedures at different centers.

We specifically investigated the effect of scanner variability by re-scanning the test sets. This shows that the performance drop can be partly attributed to scanner variation, as re-scanning the IT2 slides on the Philips scanner results in a reduction of the drop from 15 to 10 %. On IT1 the re-scanning has less impact, with slightly worse AUC (0.92–0.91), but better accuracy (0.83–0.87) for the re-scanned slides. As far as we know, this is the first study where the influence of the scanning system was explicitly investigated. Overall, our method is relatively robust to scanner differences.

Normalization is a popular pre-processing step used to transform input data to the domain of training data. In this study, we compare color and style normalization methods and their influence on the performance of deep convolutional neural networks. The AUC of the color normalization method developed by Ehtseshami Bejnordi et al., we can observe an 0.04 results improvement for IT1 and 0.02 results deterioration for IT2. For the cycle-GAN normalization, which can correct both color and style (e.g. blurring/sharpening), we can observe a large improvement in AUC, in the range of 0.06–0.14 for both test sets. Visual inspection of results, shows that cycle-GAN normalization reduces the number of false positive detections. This is also evidenced by the increased specificity at a sensitivity of 1.0 (Table 2). These results highlight the following: (a) data augmentation and multi-center training data alone do not address all sources of bias in a trained model, (b) normalization as a pre-processing step can significantly improve algorithm output, (c) full style normalization allows for a more accurate slide normalization compared to only using color normalization. However, one should always take into account that pre-processing steps such as normalization require extra processing time and might not always be the best solution.

After cycle-GAN normalization the AUC results for both independent sets (0.97 and 0.98) are in line with the results for the cross-validation results on the development set (0.98). This shows that the generalization gap that we see, and was also reported in^17^, can be closed using appropriate pre-processing. The benefit of a cycle-Gan style normalization is the possibility to retrain and adjust to the new dataset in a short time equal a few minutes, which can be reduced in the future.

In addition, our quantitative results are similar to those reported in^17^ although obtained with a smaller, supervised dataset vs. a larger unsupervised dataset. Direct comparisons are not possible due to the fact that the data is not publicly available.

In this paper we specifically focus on the task of whole-slide classification and not on segmentation of individual cancerous regions within a slide. We made this decision as our slides were not exhaustively annotated. In the future it might be interesting to specifically look into this aspect, which will also require a reference standard based on immunohistochemistry to deal with observer-variation. In addition, although cancer detection is an important first step in prostate cancer diagnostics in histopathology, future work should also address Gleason grading of biopsies specifically.

### Conclusion

In this study, the effectiveness of deep learning approaches was investigated for automatic cancer detection on hematoxylin and eosin (HE)-stained prostate biopsies. We tested three algorithms for the problem of automatic cancer detection and found that especially the U-Net approach performed better than a fully-convolutional architecture based on DenseNet and classification approach based on EfficientNet. Moreover, we evaluated the impact of the whole-slide scanners on the classification results by re-scanning the independent tests sets. Last, we investigated the effect of normalization on the output of convolutional neural networks, showing that full style normalization can improve method robustness compared to color normalization alone. Overall, the proposed system shows strong potential in pre-screening biopsies before analysis by a pathologist with a specificity ranging from 0.5 to 0.75 at 1.0 sensitivity.

## Methods

**Figure 8:**
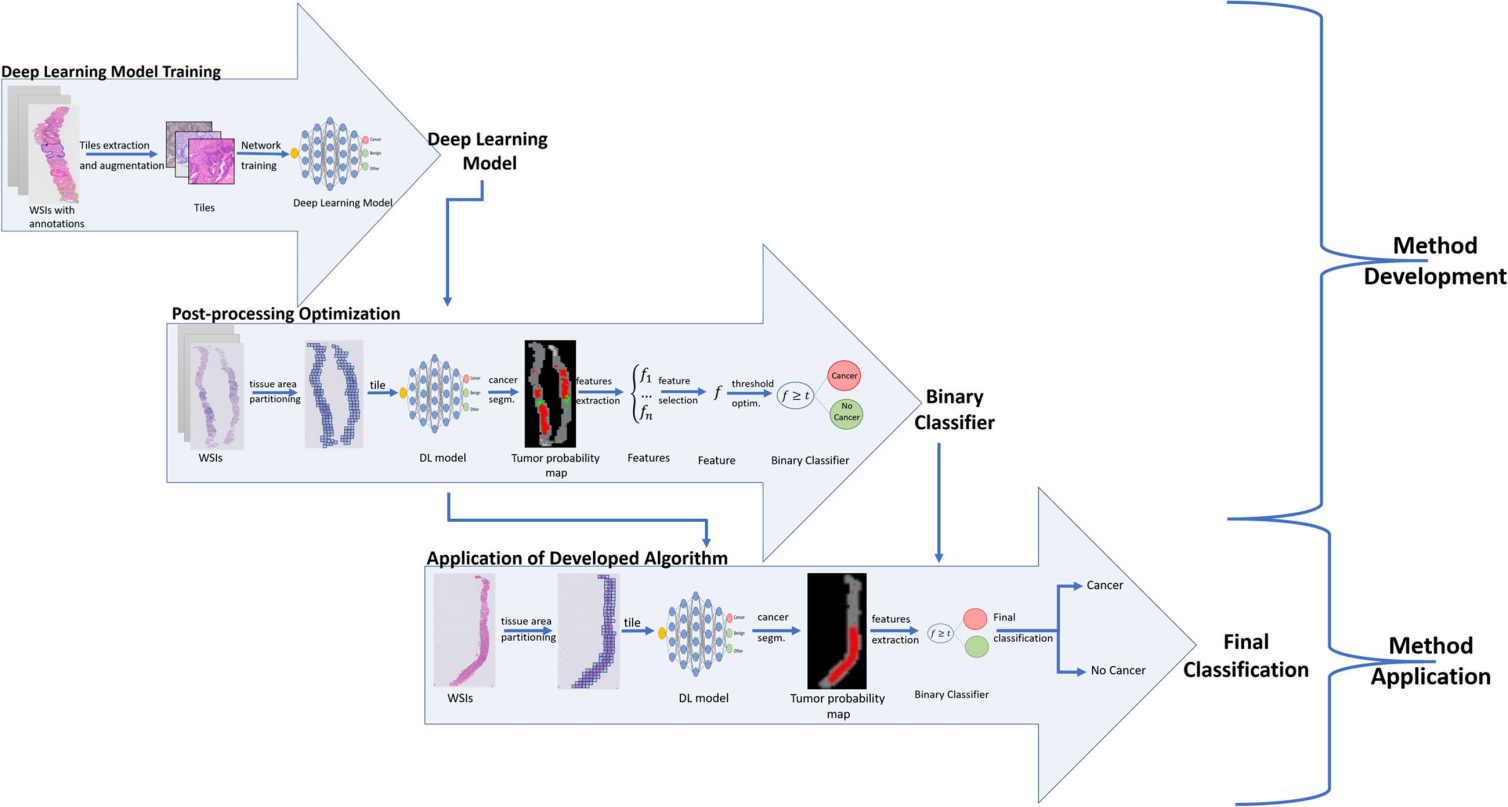
Main steps of the proposed method.

Figure 8 presents the main steps of the developed method, where we can distinguish: training of deep learning models and optimization of post-processing operations.

### Deep Learning Model Training

We investigated a deep learning strategy based on a semantic segmentation (pixel classification) by evaluating two different models, namely U-Net^20^ and DenseNetFCN^21,22^, and patch classification using EfficientNet^23^.

The U-Net model consists of two paths: a contracting path to capture context and a symmetric expanding path that enables precise localization^20^. The contraction part is the component that is mainly responsible for learning data representation, whereas the expansion part is mostly responsible for producing a fine-grained segmentation. In our study, we adapted the original U-Net^20^ architecture by increasing network depth to 5 levels to increase the context used for segmentation by adding two more blocks with 512 and 1024 filters, as well as by adding spatial dropout layers with factor 0.25 between convolutional layers, with the aim of reducing overfitting.

DenseNet^22^ is a network architecture where each layer is directly connected to every other layer in a feed-forward fashion (within each dense block). For each layer, the feature maps of all preceding layers are treated as separate inputs whereas its own feature maps are passed on as inputs to all subsequent layers. In this work we applied the fully-convolutional version of DenseNet, called DenseNetFCN, to image segmentation tasks as described in the paper^21^. The applied network has: 5 dense blocks, 16 filters added per dense block and 4 layers in each dense block.

The EfficientNet is a model proposed in 2019^23^. It is a lightweight convolutional neural network architecture achieving the state-of-the-art accuracy on ImageNet datasets. This model is based on a novel model scaling method that uses a simple yet highly effective compound coefficient to scale up CNNs in a more structured manner. The novelty of this method is uniformly scaling each network dimensions with a fixed set of scaling coefficients, based on recent progress on AutoML. In our study, we used the original EfficientNetB0 model, where weights were initialized as *“noisy-student”*^24^.

The presented models were optimized using stochastic gradient descent with a categorical cross entropy loss function. The batch size was set to 3, and the training was performed with a learning rate of 0.0005. The input patch size was $512\times 512$ pixels for U-Net model, and it was reduced to $256\times 256$ pixels for DenseNet model (patches were resized) due to memory constraints. Patches with size $512\times 512$ were extracted from images at $5\,\times$ magnification.

The segmentation problem was formulated as a pixel level multi-class problem. Due to the sparse annotations, for each extracted tile, a target map including a single class was created. Patches were automatically extracted from annotated areas, that were prepared by medical experts. Patches were selected such that they fit fully in the annotated areas. Due to the lack of reference standard labels for non-annotated areas, patches outside of annotation were not used. The number of patches extracted from a single WSI depends on the size of annotated areas. The final number of training and validation patches was equal to 1,246,629 and 221,576 respectively. The prepared target maps are used in the learning procedure of both U-Net and DenseNetFCN models. The network was trained with multiple classes in order help the network deal with difficult benign mimickers of cancers. For example, high-grade PIN areas can be very similar to cancer areas and can be easily confused. In order to reduce the risk of misclassification, we decided to use a multi-class training strategy. However, in the final validation, all non-cancer classes were grouped together.

We used data augmentation to ensure robustness to known variations in histopathology, such as rotations and color differences. This improves the robustness and ability of CNN to generalize, and decreases the risk of overfitting^25^. We applied augmentation based on a modification of brightness, contrast, saturation, and rotation, as well as additive Gaussian noise and Gaussian blur augmentation^25^. Augmentation has been applied in varying amounts for each class, where classes with fewer samples were more heavily augmented. This strategy reduces class imbalance.

In the case of the test set slides, a whole WSI was divided into patches and all patches that include tissue were classified. Patches without tissue were not classified, because they do not include the biological information.

### Post-processing

A set of features was calculated from the tumor likelihood map generated in the previous step, to establish the slide-level prediction. The following features were calculated: a 10-bin cumulative histogram of the tumor likelihood map, size of the total detected tumor area and first-order statistics on the likelihood map such as standard deviation, variance and mean. Next, the Minimum Redundancy Maximum Relevance (MRMR) feature selection technique^26^ was applied to select the most relevant features for the classification task. Based on this, the feature tumor07 (T07) was selected as the most expressive feature, where T07 is defined as:

$$\begin{aligned} T07=\frac{\sum (TPM \ge 0.7 )}{\sum TA} \end{aligned}$$

where TPM—probability map for the tumor class, where each pixel has a probability of being tumor, TA—tumor area—all pixels that got a higher probability for the tumor class, than any other class.

The slide-level labels for a dataset can then be obtained by thresholding T07 at various levels. The entire post-processing pipeline was optimized on the post-processing tuning set without using any of the slides from the development set.

We also investigated using supervised traditional machine learning techniques on top of the extracted features, such as support vector machine (SVM), random forests, and the XGB classifier^27,28^. However, they quickly overfitted to the post-processing tuning set. The application of a proposed single feature approach is more robust across the different test sets.

### Slide normalization

We can observe significant differences in the appearance of slides scanned by scanning systems from various vendors. There can be visible differences in colors and in style, where structures can be sharper or blurrier. This is a direct result of proprietary post-processing steps applied in scanning systems, that can include various filtering operations.

A successful approach to deal with this problem is the application of a pre-processing step to normalize slides. The basic strategy is based on a color normalization, in order to transfer new images to the color domain of a development set. In the present study, the Ehteshami et al.^19^ color normalization method (WSICS) was applied. A more recent alternative color normalization can be achieved by an application of a cycle-GAN network, that allows for modifications of colors and structure look (blurring/sharping)^29^. This method was inspired by the cycle-GAN application in computer vision to transfer images from one domain to another one (e.g. a photograph to a Van Gogh style painting)^30,31^. In order to apply style normalization, we used a cycle-GAN setup to facilitate unpaired image-to-image translation. A key advantage is that the cycle-GAN approach is not limited to color variations, but can also address changes such as sharpening or blurring of the image.

### Cycle-GAN

Our cycle-GAN setup generally follows the original paper^30^. For the generator architecture, we changed to a U-Net architecture, as it has been shown to work well with normalizing histopathological data^32^. The weights of the cycle-consistency loss and the discriminator loss were set to 10.0 and 1.0, respectively. Because the cycle-GAN is only able to transform from a single domain to another, we executed a separate training run for both independent test sets.

In order to train the Cycle-GAN, we randomly picked five slides from the development set and the independent test sets. In our applications, we used slides from two sets to facilitate the domain transformation. The algorithm learns to transform the stain from one set to the other and vice versa. The Cycle-GAN application allows us to change not only color intensity but allows for introducing blurring/sharping, resulting of an input image more similar to the images in the target set. Patches with size $256\times 256$ at $10\,\times$ magnification were randomly sampled from the selected slides during training. To accommodate for the low amount of patches, we created tissue background masks to allow sampling from all tissue locations in the slide (single WSI has a size in a range of 20k $\times$ 10k pixels to 85k $\times$ 200k pixels). Furthermore, we used rotation, mirroring, and scaling augmentations to further increase variety. We trained for 150 epochs, which consisted of 50 iterations with a batch size of 4. The learning rate was initially put at 0.0005 and reduced with a factor of 0.5 each time 20 epochs passed. The trained networks were applied on the whole slide images of the independent test sets using a sliding window approach, according to^32^.

## Materials

### Whole-slide images

**Figure 9:**
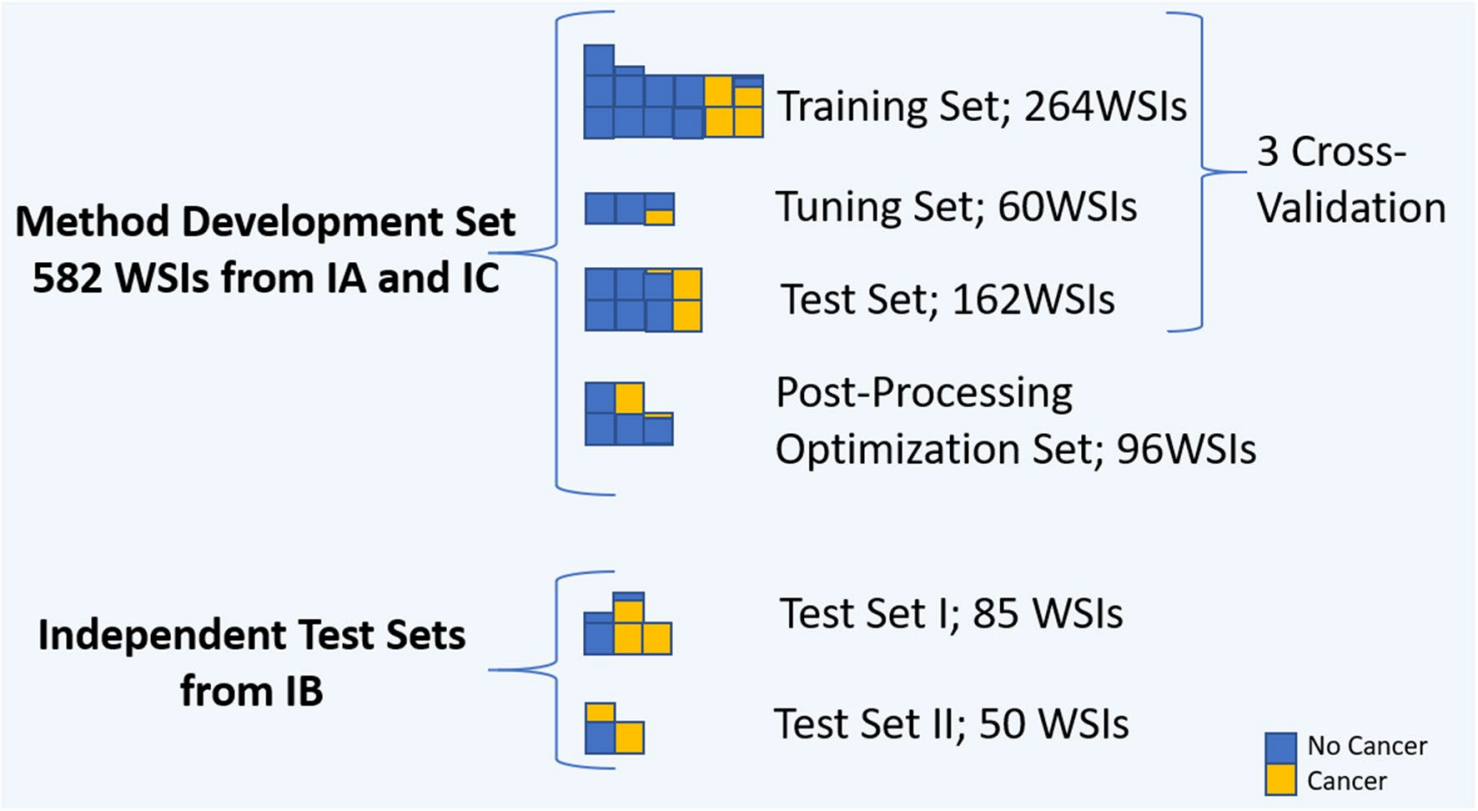
Distribution of the data used in the study across the different subsets.

For this study, we collected 717 WSIs of prostate biopsies from three medical centres in the USA (Institution C—The Penn State Health Department of Pathology, denoted as IC) and in the Netherlands (Institution A—PAMM Laboratorium voor Pathologie, denoted as IA, Institution B—Radboud University Medical Center, denoted as IB). 582 slides from two institutions (IA and IC) were used for method development, whereas 135 slides from IB were used as independent test sets 1 (IT1) and 2 (IT2) dependent on the scanner they were scanned with (see Fig. 9 and Table 3). The slides used for method development include ~25% slides with cancer, ~ 25% slides without cancer from patients with cancer and ~ 50% slides from patients without cancer, and were divided into four sets: (a) training set (264 WSIs), (b) tuning set (validation set, 60 WSIs), (c) post-processing optimization set, that not participated in the model training procedure (96 WSIs), and (c) test set (162 WSIs). Slides were stratified at to the slide-level label (cancer/no cancer) and each subset contains   25% of slides with cancer. The three-cross-validation procedure was used for the DL model training. The post-processing optimization procedure was conducted with slides (post-processing optimization set) that not participated in the previous steps of method development. We do not use the whole training set for the post-processing optimization step, because slides that were used in the DL model training can achieve a higher probability (confidence) level that unknown slides. In order to achieve high method robustness for independent (unknown) slides, this step was performed with a dedicated set of data. IT1 include 85 WSIs, where 58 WSIs contained cancer and 27 cases were negative. IT2 includes 50 WSIs, where 32 contained cancer.

All slides were stained with hematoxylin and eosin (HE). In order to keep stain variability, tissue samples were stained in the local lab of each participating medical center. Herewith, we covered a range of staining protocols (Fig. 1). Glass slides were scanned by three different scanning systems (Table 3): (a) Philips Ultra Fast Scanner (Philips, the Netherlands)—method development data, (b) Panoramic 250 Flash II scanner (3DHistech, Hungary)—independent test I (IT1), and (c) Olympus VS120-S5 (Olympus, Japan)—independent test set II (IT2). Moreover, slides from both independent test sets were rescanned on the Philips Ultra Fast Scanner.

In digital pathology there is no standard for data digitization. As a result, each scanner includes its own post-processing methods, such as an application of various filters and can have slightly different pixel sizes e.g., for a objective magnification of 20x pixel sizes can be in range 0.16–0.25 $\upmu m$. Moreover, some scanning parameters can be modified by users. This can cause the same slide scanned on the same scanner type to look different.

Our experiments were conducted with archival tissue materials obtained through standard care. Slides are fully anonymized and any patient information was not collated or stored. All necessary permission for obtaining either slides or digital images were collected from all institutions.

**Table 3:** Scanners.

Data set	Data origin	Scanners	Spatial resolution	Rescanning—scanner
Method development set	Institution A and Institution C	Philips ultrafast	0.24 $\upmu \mathrm{m/px}$	–
Independent test set 1 (IT1)	Institution B	Panoramic 250 Flash II (3DHistech)	0.24 $\upmu \mathrm{m/px}$	Philips ultrafast
Independent test set 2 (IT2)	Institution B	Olympus VS120-S5	0.16 $\upmu \mathrm{m/px}$	Philips ultrafast

Parameters of scanning systems.

**Figure 10:**
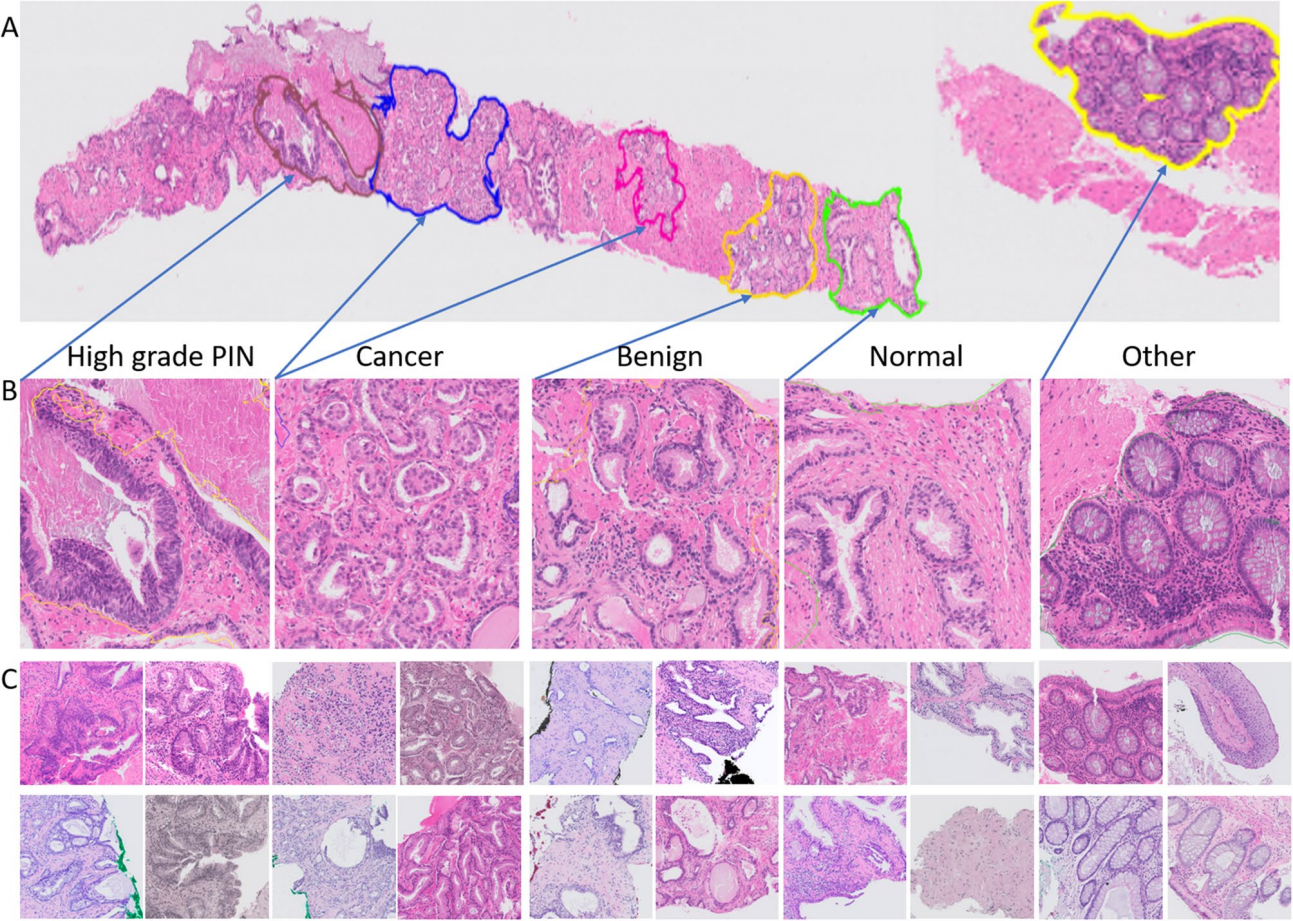
Example of whole slide images with annotations and extracted patches, where (**A**)—WSI with annotations, (**B**)—zooming of annotated areas, (**C**)—example of extracted patches for each of class. The figure was created using ASAP^34^ software, ver. 1.9.0 https://github.com/computationalpathologygroup/ASAP.

### Annotations and Class Definition

In order to develop the deep learning method, all 582 slides in the development set were manually annotated by pathologists. Annotations were made non-exhaustively in an adapted version of the open-source QuPath software^33^. Five classes were distinguished: (a) cancer (adenocarcinoma), (b) benign areas, (c) other tissue types (e.g. colon tissue), (d) high-grade PIN areas, and (e) other tissue areas. At least one area of cancer was annotated in all cases containing malignancy. Figure 10 presents an example of annotations. It should be noted that significant class imbalance occurred (especially for the high-grade PIN class, that is less represented than the other classes). Slide level labels were assigned based on the presence of cancer.
